# Assessment of the professional dental cleaning knowledge, behavior and medical compliance among dentists, medical doctors and non-medical staffs: a cross sectional study in Chongqing, China

**DOI:** 10.1186/s12903-022-02226-x

**Published:** 2022-05-19

**Authors:** Qian Wang, Hengzhu Chen, Lin Jiang

**Affiliations:** 1 College of Stomatology, Chongqing Medical University, Chongqing, People’s Republic of China; 2 Chongqing Key Laboratory for Oral Diseases and Biomedical Sciences, College of Stomatology, Chongqing Medical University, Chongqing, People’s Republic of China

**Keywords:** The professional dental cleaning (PDC), Dentists, Medical doctors, Non-medical staffs, Oral health

## Abstract

**Background:**

The professional dental cleaning (PDC) is an effective supplement that can make up for the lack of self-oral health care. Everyone should develop the habit of regular PDC. This study aimed to investigate the knowledge, behaviors and medical compliance of dentists, medical doctors and non-medical staffs about the PDC, identify the gaps, and provide information to help individuals develop healthy oral hygiene habit.

**Materials and methods:**

A web-based survey with 21 questions designed based on the characteristics, the PDC knowledge, behaviors, and medical compliance of respondents. A cross-sectional study was conducted in the main districts of Chongqing, China, in the period of September–November 2020. A total of 456 respondents including 153 dentists, 137 medical doctors and 166 non-medical staffs in 3 communities and 4 hospitals were sampled online with multistage sampling and surveyed. The data was analyzed by chi-square test using IBM SPSS Statistics v. 21.0.

**Results:**

The cognitive level and behavior of dentists on the PDC was significantly better than that of medical doctors and non-medical staffs (*p* = 0.000–0.044). The rates of not knowing “dental plaque” were 32.85% and 61.45%, of the medical doctors and non-medical staffs. Meanwhile, most of them had misunderstanding about effects of the PDC. They thought it would whiten teeth (72.99%, 80.72%), damage gums (16.79%, 19.88%) and teeth (15.33%, 21.69%), and create bigger gaps between teeth (24.82%, 33.13%). In terms of gum health and the PDC behavior, significant differences were observed, 23.53% of dentists experienced irritated gum bleeding in the last 12 months, 42.28% did not have their teeth cleaned professionally in the last 12 months, even 7.84% never had it before. Compared with it, in the same situation, the ratios of medical doctors and non-medical staffs were about 60% experiencing irritated gum bleeding, 69.34% and 77.71% not experiencing the PDC in the last 12 months, up to 33.58% and 45.18% never experienced it before. 41 respondents (8.99%) disagreed with the reasonable and necessary medical behaviors before the PDC.

**Conclusion:**

People's understanding about the PDC was insufficient, and many people, included some dentists, had not developed the habit of regular dental cleaning. Our study provided a new understanding of the PDC among dentists, medical doctors, and non-medical staffs, which may help to improve their awareness and behavior of oral hygiene health, and establish the multidisciplinary collaborations between dentists and medical doctors.

## Background

Periodontal disease (PD) including gingivitis and periodontitis is characterized by chronic inflammation. It could affect not only oral health but also systemic health. When inflammation becomes deregulated and persistent, PD may result in a systemic pro-inflammatory state which associated with an increased risk of developing cardiovascular disease, chronic respiratory disease, metabolic disease, and mental ill health. These chronic diseases are heavy burden on public health worldwide. Consequently, prevention of PD is an effective way reducing the public health burden [1–4]. PD is particularly pervasive in adults across the world [3, 5–7]. In our country, according to the fourth Chinese Oral Health Epidemiological Survey, most Chinese troubled with it. Among them, only 9.10%, 5.00% and 9.30%, of people aged 35–44, 55–64 and 65–74, had health periodontium respectively. The survey population suffered from varying degrees of gingival bleeding, periodontal pocket, and attachment loss. The detection rate of calculus exceeded 90% [8].

Poor oral hygiene is the main cause of PD and also a marker for poor health-related quality of life (HRQoL) [9]. Some studies showed the association between lower HRQoL and a higher likelihood of probable depression as well as anxiety [10–12]. The maintenance of oral health requires the combination of self-oral health behavior and professional oral care measures. The professional dental cleaning (PDC) is an effective way to keep oral health as a supplement to self-oral cleaning, and the primary treatment for PD. Its main principle is to remove the dental plaque deposited on the surface of teeth, and remain healthy periodontal tissue [13]. Therefore, the PDC is a cost-effective dental interventions that could reduce the incidence of chronic systematic diseases.

Improved oral health literacy and emphasis on prevention are keys for oral hygiene [3]. In terms of disease prevention and intervention, it is not sufficient only by dentists’ work. The general practitioner with relevant knowledge is the key. It is necessary to improve communication and collaboration between dental teams and other health professionals to ensure patients obtain an effective treatment plan of improving current health and reducing the future disease risk [1, 14, 15].

There are differences of the PDC knowledge and behavior among people. It is of great significance to investigate the PDC knowledge and behavior of people included dentists and medical doctors for knowing the deficiencies and how to improve. However, few studies have been conducted on it.

This study aimed to investigate the PDC knowledge, behaviors and medical compliance among dentists, medical doctors and non-medical staffs in Chongqing, China, and to provide information for promoting inter-disciplinary collaboration and raising oral hygiene awareness.

## Methods

### Recruitment of study participates

A cross-sectional study was conducted in the main districts of Chongqing, China from September to November 2020. Three general hospitals and the communities nearby were chosen randomly in the main districts of Chongqing, China. The dentists and medical doctors in the former, and non-medical staffs in the latter were invited to take part in the study using a self-administered questionnaire. For there were fewer dentists in general hospitals, some dentists in the Affiliated Stomatological Hospital of Chongqing Medical University were also included in this investigation. The ethics committee of this hospital approved the study and ethics committee approval number is CQHS-REC-2019 (LSNo.67). Contents of questionnaire were edited in advance on the website of “Sojump”, which is a free professional survey platform online. Then a Quick Response (QR) code was obtained. The respondents completed the questionnaires voluntarily and anonymously by scanning the QR code. The questionnaires could be filled out only once and the data was collected immediately on the website. Participants must be at least 18 years old with full civil capacity. There was a total of 456 participants in the survey.

### Study design

The self-administered questionnaire was designed for the survey. After considerable discussion by experts in preventive dentistry, the final version of the questionnaire presented. To prove the statistical validity, 15 people from each group sampled for testing prior to the formal survey. A Cronbach’s α = 0.702 was obtained, suggesting the adequate internal consistency. The demographic information of participants including gender, age, education, and occupation was requested to be filled at first (Table 1). The remaining 17 questions designed to assess the PDC knowledge, behaviors, and medical compliance of respondents. The first part was the PDC knowledge survey, including 4 questions (Table 2). In the next part, there were 6 questions about understanding of the PDC’s effects (Table 3). In the third part, gum health and the PDC behavior were studied by 4 questions (Table 4). Finally, we investigated the the medical compliance of respondents by 3 questions (Table 5).

**Table 1:** Demographic characteristics of subjects participated in the survey

	Dentists	Medical doctors	Non-medical staffs	Total
	n (%)	n (%)	n (%)	n (%)
	N = 153	N = 137	N = 166	N = 456
Gender				
Male	64 (41.83)	32 (23.36)	66 (39.76)	162 (35.53)
Female	89 (58.17)	105 (76.64)	100 (60.24)	294 (64.47)
Age				
< 30 years	73 (47.71)	34 (24.82)	64 (38.55)	171 (37.5)
30–39 years	63 (41.18)	71 (51.82)	85 (51.21)	219 (48.03)
≥ 40 years	17 (11.11)	32 (23.36)	17 (10.24)	66 (14.47)
Education				
High school degree or below	18 (11.76)	26 (18.97)	73 (43.98)	117 (25.66)
College degree or above	63 (41.18)	66 (48.18)	72 (43.37)	201 (44.08)
Master degree or above	72 (47.06)	45 (32.85)	21 (12.65)	138 (30.26)

**Table 2:** Knowledge of the PDC

Questions	Dentists n (%)	Medical doctors n (%)	Non-medical staffs n (%)	Total n (%)	Chi-square value	*P* value
	N = 153	N = 137	N = 166	N = 456		
Do you know about dental calculus?					58.544	0.000
Yes	149 (97.39)	122 (89.05)	112 (67.47)	383 (83.99)	–	–
No	4 (2.61)	15 (10.95)	54 (32.53)	73 (16.01)	–	–
Do you know about dental plaque?					145.215	0.000
Yes	149 (97.39)	92 (67.15)	64 (38.55)	305 (66.89)	–	–
No	4 (2.61)	45 (32.85)	102 (61.45)	151 (33.11)	–	–
Do you think the PDC is important for the oral health?					18.039	0.001
Yes	150 (98.04)	131 (95.62)	146 (87.95)	427 (93.64)	–	–
No	2 (1.31)	1 (0.73)	2 (1.20)	5 (1.10)	–	–
Unsure	1 (0.65)	5 (3.65)	18 (10.84)	24 (5.26)	–	–
How often do you think to do the PDC is reasonable?					115.829	0.000
6 Month–1 year	130 (84.97)	64 (46.72)	51 (30.72)	245 (53.73)	–	–
1–2 years	18 (11.76)	47 (34.31)	52 (31.33)	117 (25.66)	–	–
When needed	5 (3.27)	22 (16.06)	52 (31.33)	79 (17.32)	–	–
No need	0 (0.00)	4 (2.92)	11 (6.63)	15 (3.29)	–	–

**Table 3:** Understanding of the PDC’s effects (n/%)

Question of the PDC effects	Perspective	Dentists n (%)	Medical doctors n (%)	Non-medical staffs n (%)	Total n (%)	Chi-square value	*P* value
		N = 153	N = 137	N = 166	N = 456		
Eliminate bad breath	Right	133 (86.93)	119 (86.86)	130 (78.31)	382 (83.77)	9.727	0.044
	Wrong	7 (4.58)	8 (5.84)	22 (13.25)	37 (8.11)		
	Unsure	13 (8.50)	10 (7.3)	14 (8.43)	37 (8.11)		
Whiten teeth	Right	29 (18.95)	100 (72.99)	134 (80.72)	263 (57.68)	155.013	0.000
	Wrong	116 (75.82)	29 (21.17)	24 (14.46)	169 (37.06)		
	Unsure	8 (5.23)	8 (5.84)	8 (4.82)	24 (5.26)		
Prevent bleeding gums	Right	148 (96.73)	116 (84.67)	116 (69.88)	380 (83.33)	44.714	0.000
	Wrong	4 (2.61)	12 (8.76)	20(12.05)	36 (7.89)		
	Unsure	1 (0.65)	9 (6.57)	30 (18.07)	40 (8.77)		
Create bigger gaps between teeth	Right	8 (5.23)	34 (24.82)	55 (33.13)	97 (21.27)	106.772	0.000
	Wrong	145 (94.77)	81 (59.12)	67 (40.36)	293 (64.25)		
	Unsure	0 (0.00)	22 (16.06)	44 (26.51)	66 (14.47)		
Damage gums	Right	8 (5.23)	23 (16.79)	33 (19.88)	64 (14.04)	62.962	0.000
	Wrong	141 (92.16)	91 (66.42)	87 (52.41)	319 (69.96)		
	Unsure	4 (2.61)	23 (16.79)	46 (27.71)	73 (16.01)		
Damage teeth	Right	6 (3.92)	21 (15.33)	36 (21.69)	63 (13.82)	62.967	0.000
	Wrong	143 (93.46)	99 (72.26)	90 (54.22)	332 (72.81)		
	Unsure	4 (2.61)	17 (12.41)	40 (24.1)	61 (13.38)

**Table 4:** Gum health and the PDC behaviors (n/%)

Questions	Dentists n (%)	Medical doctors n (%)	Non-medical staffs n (%)	Total n (%)	Chi-square value	*P* value
	N = 153	N = 137	N = 166	N = 456		
Have you experienced any bleeding gums in the last 12 months?					73.855	0.000
Irritated bleeding	36 (23.53)	84 (61.31)	108 (65.06)	228 (50)	–	–
Spontaneous bleeding	1 (0.65)	3 (2.19)	5 (3.01)	9 (1.97)	–	–
No bleeding	116 (75.82)	50 (36.5)	53 (31.93)	219 (48.03)	–	–
Have you ever had the PDC before?					55.569	0.000
Yes	141 (92.16)	91 (66.42)	91 (54.82)	323 (70.83)	–	–
No	12 (7.84)	46 (33.58)	75 (45.18)	133 (29.17)	–	–
Have you ever had the PDC in the last 12 months?					45.571	0.000
Yes	88 (57.52)	42 (30.66)	37 (22.29)	167 (36.62)	–	–
No	65 (42.48)	95 (69.34)	129 (77.71)	289 (63.38)	–	–
If the PDC is needed, which one do you prefer?					31.837	0.000
Specialized dental hospitals	117 (76.47)	76 (55.47)	96 (57.83)	289 (63.38)	–	–
Stomatological Department of general hospitals	6 (3.92)	33 (24.09)	23 (13.86)	62 (13.6)	–	–
Dental clinics	12 (7.84)	11 (8.03)	15 (9.04)	38 (8.33)	–	–
wherever is convenient	18 (11.76)	17 (12.41)	32 (19.28)	67 (14.69)	–	–

**Table 5:** Compliance with the PDC (n/%)

Questions/answers	Dentists n (%)	Medical doctors n (%)	Non-medical staffs n (%)	Total n (%)	Chi-square value	*P* value
	N = 153	N = 137	N = 166	N = 456		
Past medical history of infectious diseases be asked					9.421	0.008
Reasonable	152 (99.35)	131 (95.62)	154 (92.77)	437 (95.83)		
Unreasonable	1 (0.65)	6 (4.38)	12 (7.23)	19 (4.17)		
Laboratory examination for blood coagulation be required					13.275	0.001
Reasonable	149 (97.39)	121 (88.32)	145 (87.35)	415 (91.01)		
Unreasonable	4 (2.61)	16 (11.68)	21 (12.65)	41 (8.99)		
Laboratory examination for infectious diseases be required					18.671	0.000
Reasonable	150 (98.04)	126 (91.97)	141 (84.94)	417 (91.45)		
Unreasonable	3 (1.96)	11 (8.03)	25 (15.06)	39 (8.55)		

### Statistical analysis

IBM SPSS Statistics (version 21.0) was used to analyze the data. Frequencies were calculated for all categorical variables, and Chi-square test was used to compare between the different groups. A *p*-value < 0.05 was considered to be statistically significant.

## Results

### Subjects

In total, 456 questionnaires were collected from 153 dentists, 137 medical doctors and 166 non-medical staffs. Demographic characteristics of the subjects were shown, with 64.47% of respondents being female and 35.53% being male. Of the 456 respondents, 171 were under age of 30 years, 219 were age of 30–39 years, and 66 were 40 years or older. 74.34% of participants had a diploma of college or above (Table 1).

### The PDC knowledge

Dentists had a better knowledge of the PDC than medical doctors and non-medical staffs. 97.39% of dentists knew about dental plaque, while in medical doctors and non-medical staffs, these ratios were 67.15% and 38.55% respectively, which showed statistically significantly difference (*p* < 0.05). Remarkably, 93.64% of the participants identified that the PDC is important for the oral health, included 95.62% of medical doctors and 87.95% of non-medical staffs. But In terms of “How often do you think to do the PDC is reasonable”, only 53.73% of respondents thought it should be “6mon-1 year” (Table 2).

### The effects of the PDC

It shows statistically significant differences in the understanding of the PDC’s effects among the three groups. The cognitive level of dentists was significantly higher than the other two groups (*p* < 0.05), but there were still 3.26–24.18 percent of dentists couldn't give the right answers, of which 18.95% believed it could whiten the teeth and 5.23% were not sure about it. 24.82% of medical doctors and 33.13% of non-medical staffs believed that the PDC would increase the gap between the teeth. More than 80% of medical staffs agreed with the PDC can prevent gingival bleeding, while only 69.88% of non-medical staffs agreed with it. The question of “Damage gums/teeth” had the similar results to the above (Table 3). Thus, medical doctors and non-medical staffs had relatively limited knowledge of the PDC.

### Gum health and the PDC behaviors

Compared with the other two groups, dentists performed significantly better (*p* < 0.05) in gum health and the PDC behaviors. Although 51.97% of respondents had experienced gingival bleeding in the past 12 months, there still 29.17% had never done the PDC before, including 7.84% of dentists. While, only 36.62% of participants stated that they had done the PDC in the past 12 months, and 63.38% had not experienced, also included 42.48% of dentists. When came  to the problem of where to do the PDC, most respondents would like to choose a specialized dental hospital, followed by "wherever is convenient", then the stomatological department of general hospitals, finally dental clinics (Table 4).

### Medical compliance with the PDC

The last table shows the medical compliance of respondents. Before the PDC, 95.83% of respondents thought it was reasonable when be asked about the past medical history of infectious diseases. Similarly, more than 90% of them understood and accepted the laboratory examination. The study found that there were still significant differences of the medical compliance in different groups (p < 0.05). In the non-medical staffs group, 7.23%, 12.65% and 15.06% showed low compliance with the different medical orders (Table 5).

## Discussion

Oral diseases are closely related to overall health. It is one of the major public health burden worldwide with significant socio-economic impacts [3]. Through the fourth Chinese Oral Health Epidemiological Survey, we found that the oral health, oral hygiene literacy and behaviors of Chinese people need to be strengthened and improved, which were similar to our study findings [8]. 50% of participants experienced irritated gum bleeding in the last 12 months, that means suffered from gingivitis, but only 36.62% had done the PDC which is an effective treatment for gingivitis.

Good oral hygiene is the key to maintain oral health. People with good oral hygiene had significantly better cognition of oral health than those with poor [9]. Oral health is also closely related to quality of life, it could affect not only physical, but also mental health [9–12]. Brushing teeth is the simplest self-oral health care behavior, but many people can not achieve ideal oral hygiene for their incorrect way and frequency [16, 17]. Effective oral hygiene behavior is the crucial factor in maintaining good oral health as well, which is, in turn, associated with overall health and health-related quality of life [9, 16]. The undergraduates’ oral health behaviors of a medical college had ever been investigated and the results found that the behaviors should be strengthened among the students [18].

Regular PDC can make up for the shortcomings of self-oral health care measures, it can not only completely remove plaque and calculus onto teeth, but also keep the periodontal tissue healthy and prevent the development of PD, even tooth missing [13, 19]. Moreover, it is easier to discover some mild and undetected oral diseases during the PDC, such as relatively hidden caries, for achieving the purpose of early detection and early treatment.

The PDC has been widely implemented as a clinical oral health care measure. However, many people reject it due to lacking of cognition or the experience of dentine sensitivity in the past PDC. The study had shown that oral health education before the PDC could significantly reduce the anxiety and pain of patients, and increase the popularizing rate [20]. Some domestic scholars found the health education also could enhance communication between patients and doctors, and increase the patients’ compliance [21].

The dentists undertake the important task of oral health education. Their knowledge, awareness and behaviors of oral health will inevitably affect the public’ understanding and behaviors. This study found that dentists were obviously superior to medical doctors and non-medical staffs in knowledge and behavior of the PDC because of their professional background. But there still exists a few dentists who lacking of it.

When answering questions about the PDC knowledge, 18.95% of dentists thought it can whiten teeth, while the percentages of medical doctors and non-medical staffs were 72.99% and 80.72%, respectively. The results indicated that most people and even some dentists were confused with it. In fact, the PDC can only make the teeth appear their original color by removing tea stains, smoke spots and so on, but not whiten. The dentists with right professional knowledge could make correct explanation and guidance for patients before the PDC.

In terms of behavior, still 7.84% of dentists never had their teeth been professionally cleaned before, and 42.48% did not in the past 12 months. The results may be related to their busy working and blind self-sufficiency, though they were working in the professional place. In addition, the results in the study showed that there were still a few dentists ignoring the importance of laboratory examination before the PDC, such as infectious diseases and blood coagulation. Compared with infectious diseases, examination of blood coagulation is more likely neglected. As the oral health practitioner, dentists must understand the relationship between oral and systemic health [22]. For their words and deeds would play an exemplary role in public health, it is imperative to strengthen the continuing education of dentists.

Our study revealed that although dentists and medical doctors performed better than non-medical staffs, both of them also need to improve their knowledge and behaviors. Considering the close relationship between systemic and oral health, it is necessary for medical doctors to master optimal oral health knowledge, and collaborate with dental teams [1]. The interprofessional education and collaborative practice could strengthen health systems, improve health services and health outcomes [23]. 137 medical doctors were surveyed in this study, they showed relatively weak oral health expertise, 67.15% of them knew dental plaque, 24.82% thought the PDC could increase the gap between teeth, and 15.33% thought it could hurt teeth. Their dental cleaning behavior was not ideal, 33.58% had never have the PDC before. Their misunderstanding of dental cleaning may be the barriers.

It was reported in a study that few medical staffs fully understood dental cleaning, 11.74% partially understood it, 7.4% actively requested it, and 92.96% passively received it [24]. Therefore, it is necessary to strengthen the oral health education, emphasize the importance and necessity of the PDC to medical doctors.

Among the three groups, non-medical staff is the largest and also the focus of oral health education. The results showed most of them didn't know enough about the PDC and more than 7% did not understand the medical behaviors before it, such as inquiry about infectious disease, laboratory examination for blood coagulation and infectious diseases. These medical behaviors are related to the safety of the PDC. In the clinical practice, we found that many patients rejected the laboratory examination before the PDC, mostly because of the increased cost and time. It is urgent to strengthen the public's understanding of dental cleaning knowledge and guide them to develop the habit of regular dental cleaning in the future.

The strength of this study is that the object included doctors, especially dentists who were often overlooked. Another advantage is taking the recognition degree of doctors' behaviors before the PDC, which is closely related to systemic health. However, limitations of this study must also be taken into consideration. The findings were based on self-reporting that might be subjected to information bias. The respondents may either have under- or over-reported to the questionnaire. Finally, the sample of study was relatively small, which might not be representative in China. So it is necessary to expand the sample size, strengthen publicity and organization before survey. Further in-depth investigation may should be taken place to obtain more comprehensive and detailed data.

## Conclusion

In conclusion, this study found that there were still some dentists who could not fully understand the role of the PDC, even not develop the habit of regular PDC. The general public and other medical doctors were doing less well, many of them had never experienced the PDC before. It is imperative to strengthen public awareness of oral health and help individuals develop healthy oral habits. Dentists and medical doctors also should improve their own oral health cognition and strengthen mutual cooperation for promoting public oral health.

## Data Availability

All data analyzed during this study are included in this published article.

## Abbreviations

PD: Periodontal disease
PDC: Professional dental cleaning
HRQoL: Health-related quality of life
QR: Quick response
